# An injectable heparin-Laponite hydrogel bridge FGF4 for spinal cord injury by stabilizing microtubule and improving mitochondrial function

**DOI:** 10.7150/thno.37601

**Published:** 2019-09-21

**Authors:** Chenggui Wang, Zhe Gong, Xianpeng Huang, Jingkai Wang, Kaishun Xia, Liwei Ying, Jiawei Shu, Chao Yu, Xiaopeng Zhou, Fangcai Li, Chengzhen Liang, Qixin Chen

**Affiliations:** 1 Department of Orthopedics Surgery, 2nd Affiliated Hospital, School of Medicine, Zhejiang University, 88 Jiefang Road, Hangzhou, 310009, Zhejiang, People's Republic of China; 2 Department of orthopedics Research Institute of Zhejiang University, Hangzhou, Zhejiang, People's Republic of China

**Keywords:** Fibroblast growth factor 4 (FGF4), spinal cord injury (SCI), Laponite hydrogel, microtubule, neuro-regeneration

## Abstract

**Rationale:** Spinal cord injury (SCI) remains a critical clinical challenge. The controlled release of FGF4, a novel neuroprotective factor, from a versatile Laponite hydrogel to the injured site was a promising strategy to promote axon regeneration and motor functional recovery after SCI.

**Methods:** Characterization of Laponite, Laponite/Heparin (Lap/Hep) and Laponite/Heparin loaded with FGF4 (Lap/Hep@FGF4) hydrogels were measured by rheometer. Multiple comprehensive evaluations were used to detect motor functional recovery and the axonal rehabilitation after Lap/Hep@FGF4 treatment *in vivo* (SCI rat model). Moreover, microtubule dynamic and energy transportation, which regulated axonal regeneration was evaluated by Lap/Hep@FGF4 gel *in vitro* (primary neuron).

**Results:** FGF4 released from Lap/Hep gel locally achieves strong protection and regeneration after SCI. The Lap/Hep@FGF4 group revealed remarkable motor functional recovery and axonal regrowth after SCI through suppressing inflammatory reaction, increasing remyelination and reducing glial/fibrotic scars. Furthermore, the underlying mechanism of axonal rehabilitation were demonstrated via enhancing microtubule stability and regulating mitochondrial localization after Lap/Hep@FGF4 treatment.

**Conclusion:** This promising sustained release system provides a synergistic effective approach to enhance recovery after SCI underlying a novel mechanism of axonal rehabilitation, and shows a translational prospect for the clinical treatment of SCI.

## Introduction

Spinal cord injury (SCI) is an intractably and grievously disabling disease, which induces sudden sensory and motor functional loss. The pathological process of SCI usually includes two primary phases: the structural disturbance caused by initial mechanical injury 1; worse still, it will be followed by several complex phenomena incorporating inflammation, apoptosis, oxidative stress and the formation of glial/fibrotic scars, which called secondary injury 2. The therapeutic strategies designed for SCI mainly focus on the following aspects including the inhibition of inflammatory reaction 3, suppression of glial/fibrotic scars formation 4, 5, promoting remyelination 6 and enhancing regeneration of axons 7; however, none of them have demonstrated satisfactory therapeutic efficacy in the treatment of SCI in clinical trials 8.

Fibroblast growth factor 4 (FGF4) is one of the powerful growth factors that is widely used for embryogenesis 9, angiogenesis 10 and stem cell differentiation 11. As the cell differentiation progresses, the cytoskeleton changes with the depolymerization and reorganization of the microtubules followed by morphological changes 12. Although it has been demonstrated that FGF4 could induce 5-HT neurons to develop in the hindbrain 13, no evidence suggests that function in mature neurons. Therefore, we have speculated that FGF4 might have the ability to stimulate the axon regeneration of neuron via regulating microtubule stabilization after spinal cord injury 14, 15. However, as a macromolecular protein, FGF4 has poor ability of penetrating into the blood spinal cord barrier (BSCB). Therefore, the FGF4 administration through either subcutaneous or intravenous injection is barely effective in SCI, which because the short half-life and loss of in situ blood circulation of growth factors 16. An anticoagulant named heparin has been certified that it could bind various proteins and peptides; moreover, the resulting complex can prevent the proteins from proteolysis and maintain their bioactivity 17. Moreover, FGF4 is a heparin binding member of the FGF family with an FGF homology domain that contains a heparin binding region near the C-terminus. Thus, it can be well combined with heparin. However, heparin-protein complexes are still soluble in water, which makes it difficult to perform 3-dimensional spatial controlled delivery *in vivo*. To exploit the unique properties of the affinity between heparin and FGF4, a novel injectable hydrogel was prepared here for controlled delivery of heparin-binding proteins *in vivo*.

Laponite XLG (Na^+^_0.7_[(Si_8_Mg_5.5_Li_0.3_)O_20_(OH)_4_]^-0.7^), a synthetic smectite clay, is a 2-dimensional (2D) nanomaterial composed of disk-shaped nanoscale particles that have a high aspect ratio with negatively charged surface and positively charged edge 18. This nanomaterial is of particular interest for tissue engineering and regenerative medicine in central nervous system at several different levels: firstly, it will aggregate when dispersed in solution due to the electrostatic adsorption between the negative surfaces and the positive edges and form a “House of Cards” structure 18; secondly, it could generate a stable nanoscale platelet dispersion with uniform particle size (25 nm diameter and 0.92 nm thickness) and a large surface area (>350 m^2^ g^-1^) 19; thirdly, it can readily biodegrades into non-toxic products, such as Na^+^, Mg^2+^, Si(OH)_4_ and Li^+^ , which were good for nerve cells 20, 21 and shows great biocompatibility 22. As a novel vehicle for delivery system, Laponite dispersion can either be used as a single component or in combination with other biomaterials such as alginate, collagen, hyaluronic acid and chitosan to form injectable nanocomposite hydrogels for drug delivery 19, 23, 24. However, drawbacks of these hydrogels are as follows: firstly, the absorption between Laponite dispersion particles and bioactive protein is so powerful that makes controlled release difficult, which is contradicted to the original purpose of continuous and stable release 19; secondly, these biomaterials mix with Laponite cannot protect the bioactivity of the proteins from proteolysis. Based on previous research, heparin can not only bind with the FGF family, but also is able to bind with Laponite. Thus, the Laponite gel should be efficient and stable for controlled delivery of FGF4 when complexed with heparin to form Lap/Hep@FGF4 gel. Moreover, this combined release system could exert a synergistic effect of biomaterials and growth factors, which would together enhance axon regeneration of neuron and promote functional recovery after spinal cord injury.

In this research, a novel injectable Lap/Hep gel containing FGF4 was designed, which formed easily by heparin-FGF4 hybrid mixed with the Laponite dispersion. The Lap/Hep@FGF4 hydrogel could maintain the bioactivity of FGF4 and release it to the injured spinal cord sustainably. Multiple comprehensive evidences, including functional assessments, histological and morphological were performed to evaluate the biological effect of the Lap/Hep@FGF4 gel both *in vivo* and *in vitro*. Importantly, the microtubule stabilization, an underlying mechanism in axonal regrowth was tested to clarify SCI pathology and regeneration in detail after Lap/Hep@FGF4 gel treatment. Overall, a novel injectable hydrogel validated a more precise and efficient delivery for FGF4 was prepared with remarkable physicochemical properties and promoting effect on the recovery of SCI.

## Material and Methods

### Preparation of Lap/Hep and Lap/Hep@FGF4 hydrogels

Synthetic layered silicate (Laponite XLG) with low heavy metals content was purchased from Sigma-Aldrich (St. Louis, MO, USA). Heparin sodium salt was obtained from Aladdin Industrial Corporation (Shanghai, China). Recombinant human FGF4 protein (Animal Free) was purchased from Abcam (ab217401, UK). Firstly, 1 g Laponite powder was dissolved in 50 mL double distilled water (ddH_2_O) by stirring for 2 h to form a transparent Laponite dispersion. Heparin sodium salt was also dissolved in ddH_2_O to obtain different heparin solutions (80, 160 and 320 mg/mL). The heparin concentration for Lap/Hep gels, 0.1 mL of the heparin solutions was separately mixed with 2.0 mL Laponite dispersion and immediately vibrated for 1 min to yield the Lap/Hep gels with concentrations (heparin + Laponite) of 3.8+19, 7.6+19 and 15.2+19 mg/mL (the weight ratio of Heparin/Laponite H/L, 1:5, 2:5, 4:5 respectively). Lap/Hep gels were then loaded with 500 ng FGF4 to prepare the Lap/Hep@FGF4 hydrogels. Particularly, the FGF4 solution was firstly mixed with the heparin solutions separately to yield three heparin-FGF4 complexes. Those mixtures were then added into the Laponite dispersion separately and manually vibrated for 1 min to form the 500 ng FGF4-loaded Lap/Hep hydrogels. Meanwhile, 500 ng FGF4-loaded Laponite gel was also prepared by adding the FGF4 solution into ddH_2_O and then mixed with the Laponite dispersion.

### Characterization of Lap/Hep and Lap/Hep@FGF4 hydrogels

Dynamic rheological measurements were performed on a rheometer (MCR302; Anton Paar, Graz, Austria). The Laponite dispersions, Lap/Hep gels and FGF4-loaded Lap/Hep gel were transferred to a parallel plate (40 mm diameter, gap distance 800 mm), and were covered by a thin lamination of mineral oil to prevent evaporation of solvent during rheometric test. Angular frequency sweep measurement was performed to evaluate viscoelastic properties of hydrogels by recording the storage or elastic (G') and loss (G'') moduli underlying angular frequency at 37 ℃ setups from 0.1 to 100 rad/s and a fixed strain at 1%. To investigate the gelation behavior of hydrogels, oscillatory time sweep measurement was conducted versus time at 37 ℃ with a frequency of 1 rad/s and a fixed strain of 1%. Furthermore, the shear thinning properties of hydrogels were performed by a shear rate sweep measurement with a function of the shear rate setup from 0.01 to 10 rad/s at 37 ℃. The injectability of the Lap/Hep and Lap/Hep@FGF4 gels was evaluated by a syringe. The observable self-healing ability of Laponite, Lap/Hep and Lap/Hep@FGF4 gels was measured by observing the hydrogels state after making a 0.8 cm central hole in the gels. The morphology of the prepared Laponite, Lap/Hep and Lap/Hep@FGF4 gels was observed by scanning electron microscopy (SEM, SU8010, Hitachi, Japan). Before observation, the hydrogels were freeze dried in each Eppendorf tube and transferred to the copper plate and coated with a thin gold film (Emitech, K550). Each sample was replicated at least three times.

### Zeta potential assay

Zeta potential measurements were performed using zetasizer (Nano-ZS90, Malvern). The Laponite dispersion was diluted to 10 mg/mL and heparin solutions were prepared with concentrations ranged from 10 to 320 mg/mL for use. Then 0.1 mL of 10-320 mg/mL heparin solutions was separately mixed with 2.0 mL of 10 mg/mL Laponite dispersion to yield a series of Lap/Hep complex suspensions. These Lap/Hep complex suspensions were comprised of heparin and Laponite with concentrations between 0.48+9.5 to 15.2+9.5 mg/mL (the weight ratio of Heparin/Laponite, H/L range from 1:20 to 8:5). The Lap/Hep complexes were prepared under this condition to avoid gelation, so that the mobility of the conductive components would not be hindered for zeta potential measurements. A Laponite dispersion at 9.5 mg/mL and a series of heparin solutions with concentrations between 0.48 and 15.2 mg/mL were also prepared for comparison. The freshly made Lap/Hep complex suspensions, Laponite dispersion and heparin solutions were immediately transferred into a cuvette for zeta potential measurements. At least three readings of each measurement were recorded.

### Controlled release experiment

To evaluate the FGF4 release kinetics of Lap/Hep gels, the 500 ng FGF4 loaded Laponite hydrogel and three Lap/Hep@FGF4 gels with different dose of heparin containing 500 ng FGF4 were prepared. For the FGF4 release test, 200 μL of the four hydrogels were added into tubes separately and centrifuged at 12000 g for 5 min, followed by adding with 200 μL normal saline and incubating at 37 ℃. At the specific time points of 1, 2, 3, 5, 7, 14, 21, 28 and 35 days, the gel tubes were centrifuged at 12000 g for 10 min and the supernatant was collected and replaced by the same volume of fresh normal saline. The concentrations of all released FGF4 in the supernatant were determined by ELISA assay according to the manufacturer's instruction (Westang System, Shanghai, China). The controlled release test was replicated three times.

### Spinal cord injury model and drug treatment

Total 84 adult female SD rats weighting from 220-250 g were obtained from the SLAC Laboratory Animal Company (Shanghai, China) for the *in vivo* experiments. All the animal procedures were approved by the Institutional Animal Care and Use Committee of Zhejiang University. All the rats were housed under controlled environmental conditions. Before the surgery, all the animals were anesthetized by 1% (w/v) pentobarbital sodium (4 mL/kg, i.p.). In order to fully expose the spinal cord, a laminectomy at the T9 vertebrae was performed followed by a layer-by-layer incision through the skin, subcutaneous tissues and fascia to separate the lateral muscular tissues. Afterwards, a moderate crushing injury was implemented using a vascular clip for 1 min (30 g forces, Oscar, China) to induce a spinal cord injury (SCI) model of rats 25. Immediately, 10 µl of normal saline, Lap/Hep hydrogel, a free FGF4 solution (500 ng) or Lap/Hep hydrogel containing 500 ng FGF4 was orthotopically injected using a micro-syringe (26-gauge) to cover the injured site. The rats of SCI group received the same surgical procedure but were treated with 10 µl normal saline. The rats of sham group underwent a laminectomy but sustained no crushing injury, though the spinal cord was left exposed for 1 min. Postoperative treatments included injection of penicillin solution (4 × 10^6^ units per animal, i.p.) in the first five days and manual bladder emptying twice a day. Subsequently, the rats were sacrificed and the spinal samples were harvest at 14 and 28 d after treatment.

### Functional behavior evaluation

Hind-limb motor function was evaluated using the Basso-Beattie-Bresnahan (BBB) locomotion scale and the footprint test at 0, 1, 3, 7, 14, 21 and 28 day post-SCI. Briefly, the BBB locomotion rating scale ranges from 0 (complete hind limb paralysis) to 21 (normal locomotion) points based on walking ability, the movements of hindlimb joints, and coordination 26. Each rat was observed separately for 5 min per session and their BBB scores were recorded. Footprint analysis was performed by dipping the animal's posterior limb with blue dye and fore limb with red dye 27. The animal was then allowed to walk across a narrow pipeline (90 cm in length and 10 cm in width). The footprints were scanned, and the resulting digitized images were analyzed. The outcome measures were obtained by three independent examiners who were blinded to the experimental conditions.

### Western blot

Cells and 100 mg of spinal cord tissue from rats at the contusion epicenter were washed 3 times with ice-cold PBS and lysed in RIPA buffer containing 1% PMSF. Then samples were centrifuged at 12500 g for 30 min at 4 ^°^C. Protein concentrations in supernatant were assessed using the bicinchoninic acid protein assay kit (Beyotime, Shanghai, China). Equal amounts of protein extracts were resolved by 4%-20% SDS-PAGE and electro-transferred onto the polyvinylidene difluoride membranes (Millipore, Bedford, MA). After blockade in Tris-buffered saline plus 5% (w/v) milk (Bio-Rad), membranes were exposed to primary antibodies: GFAP (1:2000, Abcam), NF-200 (1:2000, Abcam), microtubule-associated protein 2 (MAP-2, 1:1000, Cell Signaling Technologies), NG-2 (1:1000, Abcam), Laminin (1:1000, Abcam), Neurocan (1:1000, Abcam), MBP (1:1000, Abcam), CD68 (1:1000, Abcam), Ace-tubulin (1:1000, Abcam), Detyrosinated tubulin (1:500, Proteintech, China), Tyrosinated tubulin (1:500, Proteintech, China) and GAPDH (1:1000, Cell Signaling Technologies), in the blocking solution overnight at 4 ^°^C. Horseradish peroxidase-conjugated rabbit/mouse-specific antibodies were incubated with samples for 60 min at room temperature. Signals were visualized by Chemi DocXRS + Imaging System (Bio-Rad), and ImageJ software was used to test gray levels. All experiments were repeated three times.

### Statistical analysis

All values are presented as the mean ± SD. Statistical differences among groups were analyzed using one-way analysis of variance (ANOVA) followed by Tukey's post hoc test. Differences between groups in BBB scores were detected using repeated measurement two-way mixed ANOVA, followed by Tukey test to detect differences between groups. When the values in the study were not normally distributed, statistical differences were analyzed using one-way ANOVA on ranks with post hoc Dunn's method. In all the analyses, *P < 0.05 and **P < 0.01 were considered statistically significant.

## Results and discussion

### The characteristic of Laponite, Lap/Hep and Lap/Hep@FGF4 gels

**Figure 1:**
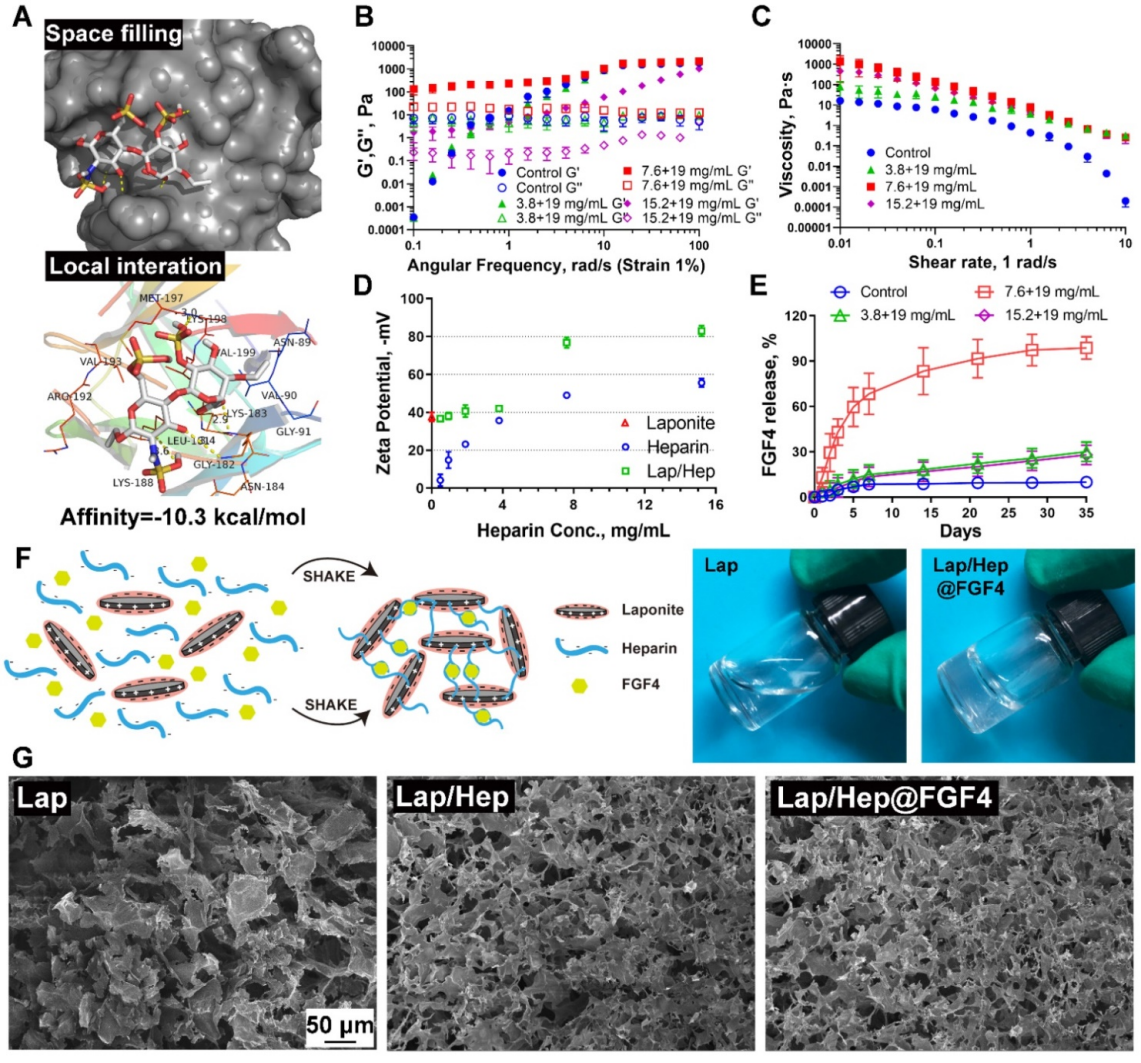
** Characterization of Laponite, Lap/Hep and Lap/Hep@FGF4 hydrogels.** (A) Docking studies were performed as described in Materials and methods. The protein residues are shown in a ribbon model. The space filling models show the binding of heparin monomer in the FGF4 binding pockets. (B) Angular frequency sweep measurements of Laponite and Lap/Hep gels. (C) Viscosity change of Laponite and Lap/Hep gels with different concentrations as a function of shear rate. (D) Zeta potential measurements of Laponite, heparin and Lap/Hep as a function of heparin concentration. (E) Cumulative release of FGF4 from Laponite and Lap/Hep gels. (F) Schematic illustration of gelation between heparin-FGF4 complex and Laponite to form Lap/Hep@FGF4 gel. Once the two components are mixed with manually shaking, the whole solution turns into transparent gel. (G) SEM images of the lyophilized Laponite, Lap/Hep and Lap/Hep@FGF4 hydrogels. All experiments were performed in triplicate and data are presented as Mean ± SD.

Clinically, the low therapeutic efficacy of free growth factors may attribute to their rapidly biodegradable rates by proteolysis *in vivo*. Therefore, mass of research has showed growth factors combined with heparin could form a stable structure to prevent proteolytic cleavage, which promote the bioactivity of growth factors; however, the water solubility of heparin-growth factors is still a hard nut to crack 28, 29. To examine whether heparin has a favorable affinity to FGF4, we performed a molecular docking analysis of a heparin monomer with FGF4 according to diverse binding pockets. By studying all the models returned, we found that heparin formed some favorable connections and docked nicely within FGF4 binding sites with a high affinity of -10.3 kcal mol^-1^, and the space filling models are used to directly illustrate the coverage of heparin in the related protein structures. Meanwhile, the macro views and the image of the local interactions of protein residues are shown in a ribbon model (Figure 1A). Taking advantages and disadvantages of the heparin-growth factors complex into consideration, our group designed a novel Lap/Hep hydrogel for local delivery of growth factors via blending heparin-growth factors complex and Laponite dispersion together. Pervious researches have showed that the mechanical properties of the hydrogels are the main factors affecting cell survival, neuronal differentiation, and axon formation 30-32. To investigate the mechanical properties between the composition, 19.5 mg/mL of Laponite dispersion was chose as control and various dose of heparin solution or heparin-FGF4 complex were mixed with control Laponite dispersion to yield Lap/Hep gel with concentrations ranging from 3.8+19, 7.6+19 and 15.2+19 mg/mL (H/L, 1:5, 2:5, 4:5) or 7.6+19 mg/mL Lap/Hep gel containing 500 ng FGF4.

Viscoelastic properties and gelation kinetics were evaluated by dynamic rheological measurements, which revealed the shear thinning and injectable properties of the Lap/Hep gel. Specifically, angular frequency sweep measurements demonstrate that the Lap/Hep gels with concentrations of 7.6+19 (H/L, 2:5) and 15.2+19 (H/L, 4:5) mg/mL have higher G' values than G'' values throughout the frequency sweep from 0.1 to 100 rad/s (Figure 1B); moreover, the G' values of control Laponite and 3.8+19 mg/mL (H/L, 1:5) Lap/Hep hydrogel increased throughout the frequency sweep and surpassed the G'' values at around 1 rad/s angular frequency, which demonstrated that those dispersion can form a weak gel through face-to-edge interaction when angler frequency increase. Moreover, the 7.6+19 mg/mL (H/L, 2:5) Lap/Hep gel showed higher G' and G'' values than the 15.2+19 mg/mL (H/L, 4:5) gel, which demonstrated that the concentration of 7.6+19 mg/mL (H/L, 2:5) Lap/Hep gel may possess good elastic property, and will not be easily destroyed under appropriate shear stress through the quick reversible interactions. Moreover, the G' value (elastic moduli) of Lap/Hep hydrogels at the concentration of 7.6+19 mg/mL (H/L, 2:5) that highly matched with spinal cord tissue (0.1-16 kPa) can better imitate real tissue for use in SCI models 32, 33. A shear rate sweep measurement was implemented to confirm the shear thinning behavior of the Lap/Hep gels for injectability (Figure 1C). Furthermore, the optimal concentration of heparin has confirmed by Zeta potential to examine the change of charge upon mixing the two components. In this case, the Laponite concentration is diluted to 10 mg/mL, so that it can complex heparin without forming a gel. The latter will hinder the mobility of conductive components for zeta potential measurement. The Lap/Hep complex suspensions have concentrations ranged from 0.48+9.5 to 15.2+9.5 mg/mL (H/L, 1:20 to 8:5). The sharp drop of zeta potential was evidently observed in Figure 1D, which implied a saturating absorption of heparin to Laponite occurred at the concentration of heparin about 3.8 mg/mL (H/L, 2:5). Above this threshold, more free heparin molecules contribute to zeta potential value in a similar trend as the heparin solution control shows. Thus, the zeta potential measurement provides a good evidence for the proposed crosslinking mechanism based on electrostatic interactions between heparin and Laponite edges and provide an optimal weight ratio of Heparin/Laponite at 2:5, which consist with rheological measurement.

The key advantage of the obtained Lap/Hep gel is that heparin can bond to the growth factors and avoid the tight connecting between growth factors and Laponite nanoplatelets. The heparin dose was set differently to compare the effects on release kinetics of FGF4. *in vitro* release profile of FGF4 from different Lap/Hep gels were collected and recorded. As shown in Figure 1E, an initial burst release (~13%) of FGF4 on day 1 and continued cumulative release over 60% within day 5 were observed in 7.6+19 mg/mL (H/L, 2:5) gel group. With the increase of time, approximately 83% and 99% of the loaded FGF4 was released from the 7.6+19 mg/mL (H/L, 2:5) hydrogel at day 14 and 35, respectively. However, a negligible amount of FGF4 at day 5 was released from other three groups, and only about 28% of FGF release can be found in both 3.8+19 mg/mL (H/L, 1:5) and 15.2+19 mg/mL (H/L, 4:5) hydrogels after day 35. Thus, a sustained release behavior of FGF4 was observed in the 7.6+19 mg/mL (H/L, 2:5) hydrogel, which could achieve spatial as well as temporal control in the delivery of FGF4. The release kinetics of FGF4 from the hydrogels primarily depends on the binding force between FGF4, heparin and nanoplatelets. Here, FGF4 is blended with heparin to form heparin-binding proteins first and then loaded in the Laponite dispersion to yield Lap/Hep@FGF4 gels (Figure 1F). When at low concentration of 3.8+19 mg/mL (H/L, 1:5), the heparin-FGF4 complex is more easily absorbed onto the positive edge of Laponite nanoplatelets via electrostatic interaction, showing a slower and steadier release compared to the one with the moderate concentration of 7.6+19 mg/mL (H/L, 2:5). Interestingly, the gel with higher concentration (15.2+19 mg/mL, H/L, 4:5) of gels results in a slower release again. This may mainly attribute to the more amount of free heparin molecules reciprocity with FGF4 to prevent release the latter within the gel, thus decreasing the release rate 34, 35. This release kinetics of FGF4 consist with the rheological and Zeta potential results (Figure 1B and 1D), indicated that the optimal H/L for FGF4 controlled release is 2:5 and the concentration of gel for bioactivity assay is 7.6+19 mg/mL. When the free FGF4 incorporated in the Laponite controls, the FGF4 molecules are rapidly interacting with the nanoplatelets through physical absorption, electrostatic interaction and hydrogen bonding, leading to the fixation of growth factors in the edge of nanoplatelets and further slowly release situation 19. Meanwhile, the G' and G'' value from time, angular frequency and shear rate sweep measurements was used to evaluate the gelation rate upon mixing with the FGF4. The three measure systems showed similar values of G' and G'' (Figure S1A-S1C) indicated that mixing FGF4 into Lap/Hep gel would not alter the gelation rate in 7.6+19 mg/mL (H/L, 2:5) gel group. Moreover, the morphology of the Laponite, Lap/Hep and Lap/Hep@FGF4 hydrogels was also observed under SEM. Compared to the loose structure of the Laponite controls, the Lap/Hep and Lap/Hep@FGF4 hydrogels had a relatively compact structure resembling a cribriform plate (Figure 1G), and possessed a 3D porous structure, which are favorable for preventing proteolysis of FGF4 and maintaining delivery of FGF4 locally and sustainably. Based on these results, the prepared Lap/Hep gel could be used as a sustainable and stable carrier for controlled delivery of FGF4. Moreover, the ability of injection of Lap/Hep and Lap/Hep@FGF4 hydrogels were certified by a syringe, which showed the Lap/Hep and Lap/Hep@FGF4 hydrogels could be extruded through a 26-gauge needle without clogging (Figure S1D). The macroscopic flow and self-healing properties of hydrogels are illustrated in Figure S1E. The Laponite, Lap/Hep and Lap/Hep@FGF4 hydrogels exhibit the properties of self-healing, as evident by the visual observation that a 0.8 cm central hole in the gel could diminish and the Lap/Hep@FGF4 gel could finally disappear after 12 h (Figure S1F). The self-healing mechanism was attributed to the reversible electrostatic interaction between nanoplates and heparin.

In this case, we expect that the Lap/Hep gel could suit for spinal cord, which is mainly attributed to the following aspects: first, it has the similar elastic moduli with CNS tissue; second, Lap/Hep gel could inject into injured site easily by syringe; third, Lap/Hep is a self-healing hydrogel, which could avoid the irreversible break of bonds between nanoplates and heparin; last, Lap/Hep gel could release FGF4 sustainably and stably.

### Lap/Hep@FGF4 improved pathology and motor function after SCI

The concentration of heparin used in Lap/Hep gels is below its clinical dosage as an anticoagulant. Moreover, heparin is bound to the edge of nanoplatelets, thus it can hardly react to other chemicals as a free monomer *in vitro* and *in vivo*. Flow cytometry was performed to further evaluate the biomaterial toxicity of Lap/Hep gels. As show in Figure S2, the Laponite control, Lap/Hep and Lap/Hep@FGF4 hydrogels showed no toxicity to neurons, which demonstrates the good biocompatibility of the gels.

**Figure 2:**
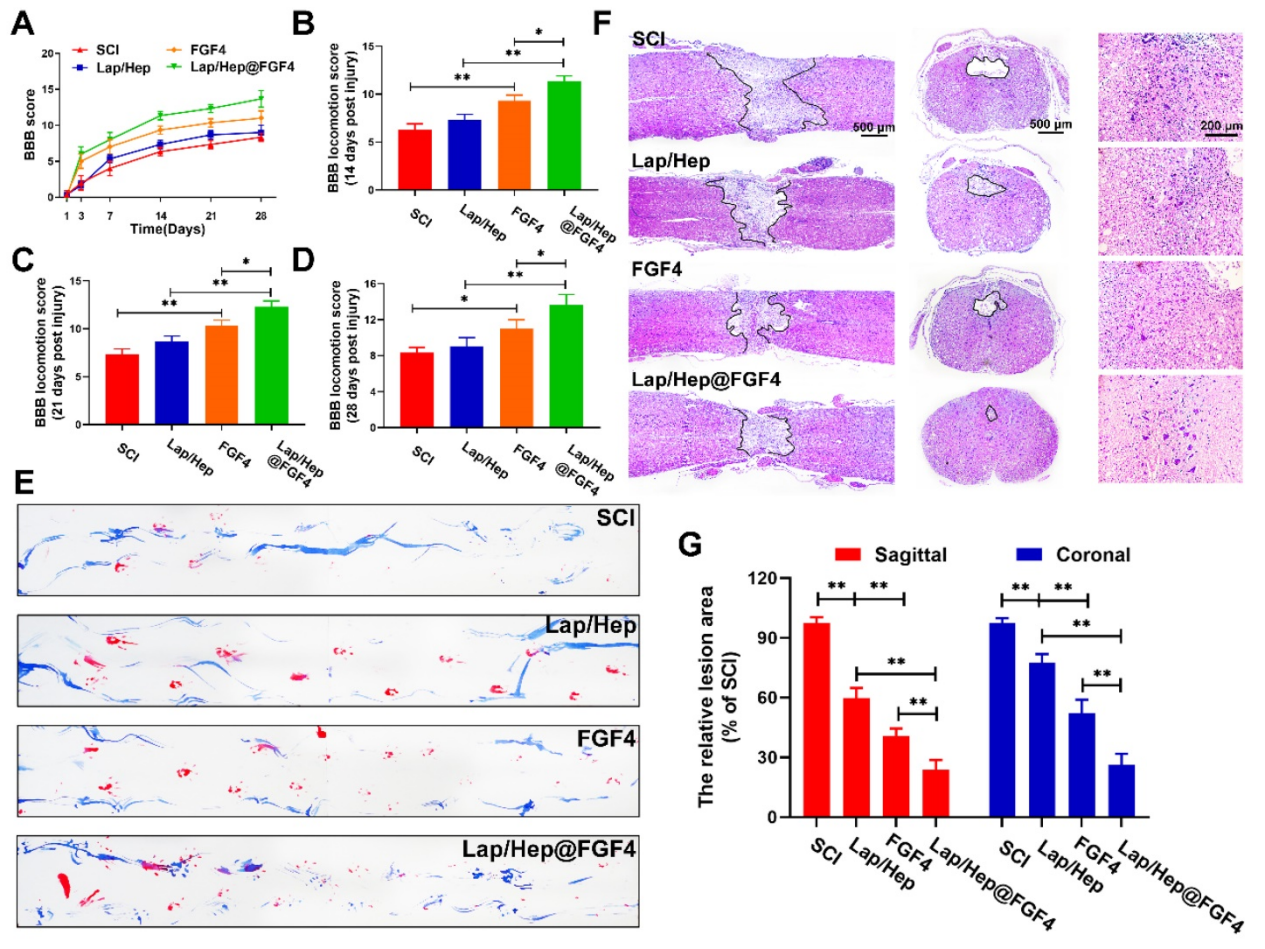
** Lap/Hep@FGF4 improves pathology and motor function after SCI.** (A) The Basso-Beattie-Bresnahan (BBB) locomotion scores of the different groups. (B-D) Quantification of BBB locomotion scales at 14, 21 and 28 days post-injury from A. (E) Footprint analyses of the different groups. (F) Representative images from H&E staining at 28 days post-injury. (G) Quantification of the lesion area of the spinal cord from H&E staining in sagittal and coronal. Values were expressed as the Mean ± SD, n= 4 per group.

Comprehensive pathological and functional measurements were performed to evaluate neural motor recovery after SCI. Functional assay composing the Basso-Beattie-Bresnahan (BBB) locomotion scores and the footprint test were used to evaluate whether the FGF4 and Lap/Hep gels could enhance motor functional recovery of rats after SCI. With the progress of spinal cord injury, the animals exhibited flaccid paralysis with followed proper recovery in a time-dependent manner in all groups (Figure 2A-2D); however, the degree of recovery varied between groups evaluated by BBB scores. Compared to the SCI group (average BBB score of 6), the BBB scores of rats that received Lap/Hep (average BBB score of 7), free FGF4 (average BBB score of 9) and Lap/Hep@FGF4 (average BBB score of 11) treatment increased after 14 days of injury. At day 21, the BBB scores of each group increased smoothly, and the BBB scores of the Lap/Hep@FGF4 group were significantly higher than other three groups. The footprint test intuitively revealed that rats in Lap/Hep@FGF4 group showed a fairly consistent posterior limb (blue ink) footprint with partial coordination and few stumbling at day 21 after SCI. Meanwhile, rats in Lap/Hep gel and free FGF4 groups showed inconsistent behavior with extensive dragging; however, compared to SCI group, the width of blue ink streaks still increased in these two groups, which can be seen as a sign of the ankle, knee, and hip joint of posterior limbs of the rats healed better and can significantly move (Figure 2E). Based on those results of functional measurements, the Lap/Hep gels used here could increase the motor functional recovery of rats after SCI even with no statistically significant BBB scores. It can be explained that the damage of spinal cord injuries may be caused by the ion balance interruption 36, while the biodegradation products of Laponite can help maintain the ion balance of the rats after treatment, thus improve the SCI recovery at a relatively small extend. Furthermore, both the free FGF4 and Lap/Hep@FGF4 gels treatment manifested remarkable promotion of the functional recovery in rats, and Lap/Hep@FGF4 gels showed the best therapeutic effect on SCI rats compared with other groups.

H&E staining showed the histological morphology in the injured spinal cord at day 28 after SCI (Figure 2F and 2G). Severely damage and an obvious cavity were observed on the injured site of the spinal cords at day 28 after SCI in both longitudinal and transverse sections. The relative lesion area in the injured spinal cords treated with Lap/Hep, free FGF4 and Lap/Hep@FGF4 significantly decreased with less damaged tissue. Moreover, the number of neurons in grey matter of injured spinal cord was remarkably enhanced by Lap/Hep@FGF4 treatment. Consistent with our results, the Lap/Hep@FGF4 treatment significantly decreased the damage of tissue and neurons in the ventricornu, and Lap/Hep gel or free FGF4 also showed therapeutic effect on SCI, indicating that the Lap/Hep gel loaded with FGF4 could exert a synergistic effect of reducing tissue loss and protecting neurons in the lesion area due to the localized and sustained delivery of FGF4, together contribute to the locomotion recovery after spinal cord injury.

### Lap/Hep@FGF4 promotes the axonal generation after SCI

**Figure 3:**
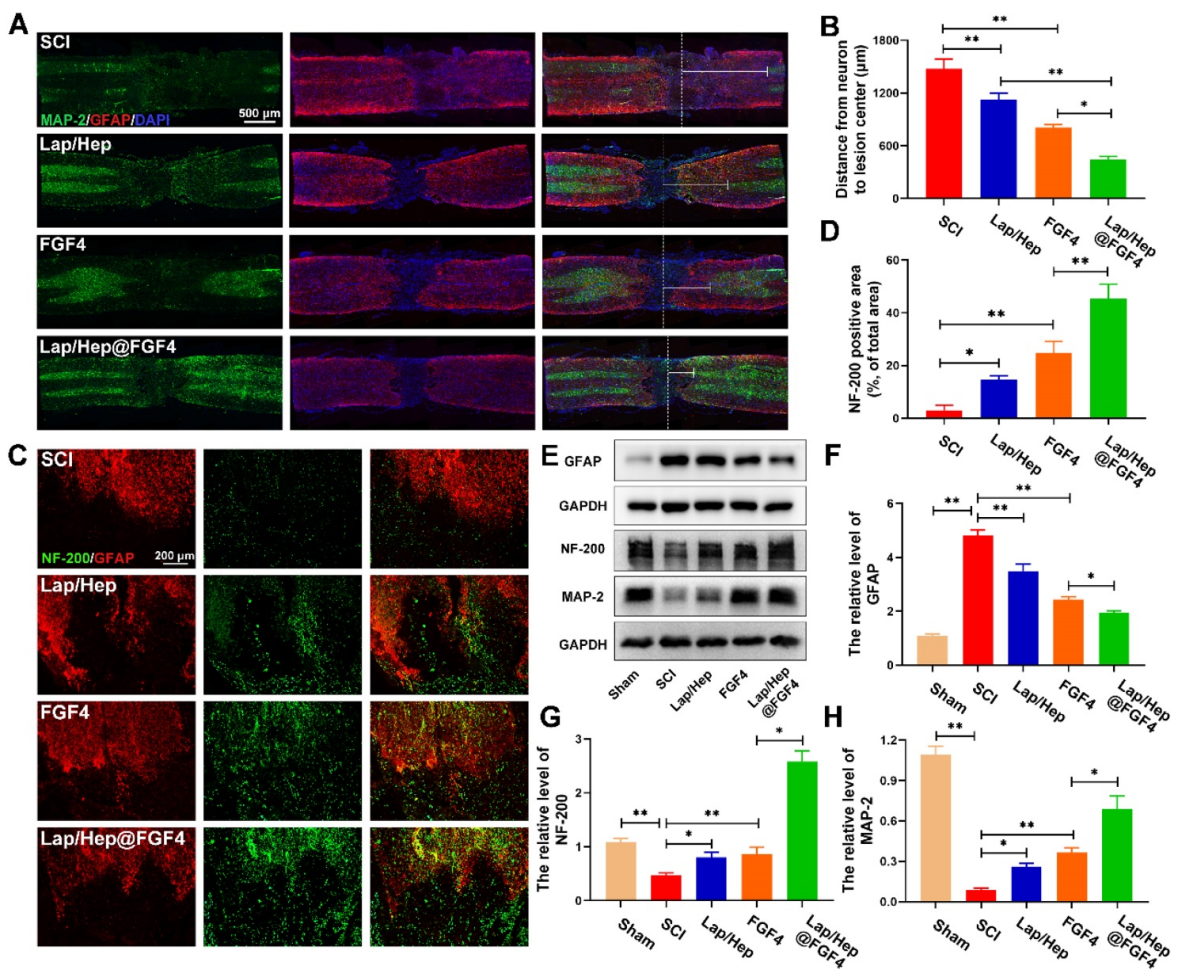
** Lap/Hep@FGF4 enhances axonal regeneration after SCI.** (A) Co‐immunofluorescence images show GFAP (red) and MAP-2 (green) at 28 days after SCI in each group, the white triangle and dotted line indicate border of lesion site. The white dash line indicated the lesion center. (B) Quantification of distance from neurons to lesion center. (C) Representative images containing astrocytic and neurofilament (NF-200) immunofluorescence on spinal cord sections at 28 days after SCI. (D) Quantitative analysis of NF-200 positive axon area in the lesion. (E-H) Western Blot (WB) protein expressions and quantification data of GFAP, NF-200 and MAP-2 in each group. All experiments were performed in triplicate and values were expressed as the Mean ± SD, n= 4 per group.

With the pathophysiology of SCI ulterior comprehend, protecting nerve and tissue has fueled a move towards to promote neurorehabilitation, neuroplasticity and axonal regeneration as potential and advanced therapeutic approaches to spinal cord injury 37, 38. Immunofluorescence and western blot were performed to evaluate the effect of axonal rehabilitation by Lap/Hep@FGF4 controlled release system using spinal cord tissue at day 28. The MAP-2, a structural protein and constituent of axon microtubules, was used to measure the distance from the nearest neurons (MAP-2 positive cells) to lesion center (white dash line) in the injured spinal cord in each group by immunofluorescence (Figure 3A). Compared with SCI group, the distance in the injured spinal cords treated with Lap/Hep, free FGF4 and Lap/Hep@FGF4 significantly decreased, indicating that all the three treatments could enhance the recovery of the destroyed axon and the Lap/Hep@FGF4 gel shows the most significant effect on promoting the axonal regeneration after SCI (Figure 3B).

The axonal regeneration is regarded as an elementary factor for the functional restoration after SCI. Double immunofluorescence staining for GFAP (red) and NF-200 (green) was performed to observe the lesion center of spinal cord and the extension of the neurofilaments (Figure 3C and 3D). In the SCI group, few NF-200 positive axons were observed in the area, whereas the Lap/Hep gel and free FGF4 groups showed moderate NF-200 positive axons with punctiform or tubules. Additionally, Lap/Hep@FGF4 treatment significantly boost the neurofilaments regeneration of the injured rats, which could traverse the barrier of glial scar and present extended axons. Moreover, the western blot showed the GFAP expression remarkable decreased by Lap/Hep@FGF4 treatment (Figure 3E and 3F), which reduced the inhibition effect of glial scar. Axonal regeneration can not only be affected by the FGF4, but also can be influenced by Lithium, which is a byproduct of Laponite degradation and have therapeutic potential in the central nervous system injury due to its ability to promote the neuronal survival and generation 39. The western blot analysis results for NF-200 and MAP-2 (Figure 3E, 3G and 3H) also revealed similar group differences trend to the immunofluorescence.

In summary, our results demonstrated that Lap/Hep gel and FGF4 could moderate promote the axonal growth; moreover, FGF4 combined with Lap/Hep gel exerted greater effects on axonal generation than single use, indicating that the novel controlled release system (Lap/Hep@FGF4) showed a synergistic effect on stimulating axonal growth.

### Lap/Hap@FGF4 reduces fibrotic scar tissue and astrocyte migration/polarization

**Figure 4:**
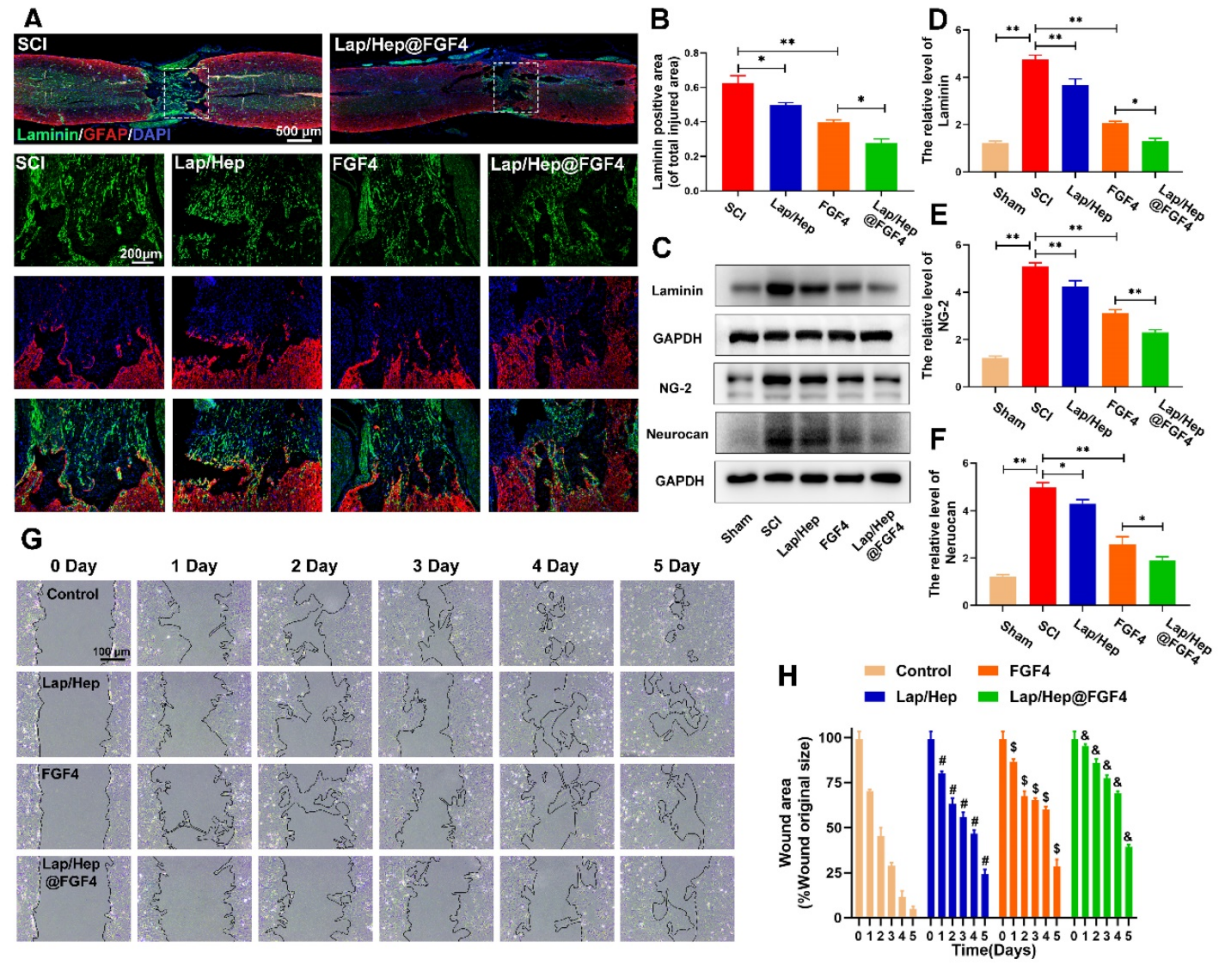
** Lap/Hep@FGF4 reduces fibrotic scar tissue formation at the injury site and suppresses astrocyte migration *in vitro*.** (A, B) Immunofluorescence staining and quantification data of laminin (green) and GFAP (red) in the spinal cord at 28 days post-injury. (C-F) Protein expression and quantification data of laminin, NG-2 and neurocan in each group. (G) Photograph of astrocyte migration regulated by Lap/Hep@FGF4 treatments at 5 days. (H) Quantification data of wound areas showed that astrocytes migrated evidently slower in the Lap/Hep@FGF4 group; # P < 0.05 versus the control group, $ P < 0.05 versus the control group, & P < 0.05 versus the free FGF4 treated group. All experiments were performed in triplicate and values were expressed as the Mean ± SD, n= 4 per group.

Seal-like scars in the lesion site are usually formed in the injured site of spinal cord and composed of cellular components and extracellular matrix (ECM). The roles of the scar after SCI are complicated and controversial 40. On the basis of their appearance, early reports stated that astrocytic scar is a barrier to axon regeneration and attenuating glial scar formation enables spontaneous axon regrowth 41, while glial scar formation aiding central nervous system axon regeneration have also been recently demonstrated 42. As similar with fibrotic scar, it contained mass of extracellular matrix, which can create a favorable microenvironment for nerve fiber regrowth 43. However, series of inhibitory factors, such as chondroitin sulfate proteoglycan (CSPGs), myelin-associated glycoproteins (MAGs) and Nogo, are excessively secreted by the hypertrophic fibrotic scar, which cause an inhibitory spinal microenvironment for axonal regrowth 44. As illustrated in Figure 4A and 4B, compared with SCI group, laminin-positive fibrotic scarring area was remarkably decreased after Lap/Hep@FGF4 treatment at day 28 post-injury. Additionally, Lap/Hep gel and free FGF4 treated groups also showed decreased laminin positive area consist with the western assay of laminin protein expression (Figure 4C and 4D). Reduction of fibrotic scar tissue by Lap/Hep@FGF4 treatment was associated with a decrease of NG-2 and neurocan (Figure 4E and 4F), which act as similar with CSPGs.

Along with the process of SCI, the astrocytes proliferate, migrate and assemble to the wound border would severely inflame and damage the spinal tissue, which last 2 or 3 weeks to form a scar border 45. A scratch assay combined with transwell co-culture system was used to investigate the astrocytes' migration influenced by Lap/Hep gel, free FGF4 and Lap/Hep@FGF4. When co-cultured with Lap/Hep gel or free FGF4, astrocytes showed a moderate reduced migration rate, while still faster than Lap/Hep@FGF4 treatment (Figure 4G). Specifically, compared with control group (4.9%), the wound area of Lap/Hep gel and free FGF4 group were remarkably decreased to 24.2% and 28.5% of the original area at day 5. Meanwhile, in Lap/Hep@FGF4 treated group, the wound area only showed a small decrease during the 5 days of observation, and still remain 39.3% wound area at day 5 (Figure 4H). Glial scar or reactive astrogliosis formation is often associated with cellular hypertrophy, glial fibrillary acidic protein upregulation, cell migration, and CSPGs secretion 46; moreover, the capacity of migration often influenced by the state of polarization 47. In wound scratch assay, free FGF4 and Lap/Hep@FGF4 inhibited the migration of astrocytes by changing their polarized state and microfilaments network (Figure 4G and S3). Control cells polarized by forming a leading edge compared with the round and non-polarized cells in FGF4 and Lap/Hep@FGF4 groups (Figure S3).

Collectively, these data suggest that Lap/Hep@FGF4 could significantly reduce the formation of fibrotic scar and remodel the glial scar through suppressing astrocytes migration and polarization. It may be attributed to the bioactivities of FGF4, which is highly maintained by the Lap/Hep gels.

### Lap/Hep@FGF4 reduces inflammatory reaction and promotes remyelination

**Figure 5:**
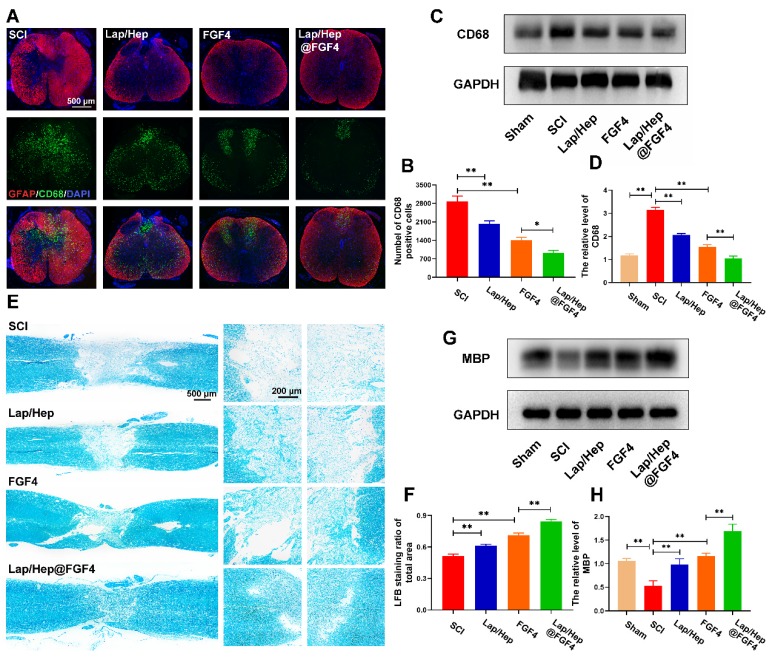
** Lap/Hep@FGF4 reduces inflammatory reaction and promotes remyelination.** (A) Immunofluorescence images of the spinal cord at 14 days post-injury shows the distribution of leukocytes (CD68, green) within the GFAP (red). (B) Quantification data of CD68 positive cells in spinal cord. (C, D) Western Blot protein expressions and quantification data of CD68 in each group. (E) Representative images of the whole spinal cord with LFB staining of the myelin sheath at 28 day post injury. (F) Quantification data of LFB positive area in spinal cord. (G, H) Western Blot protein expressions and quantification data of MBP in each group. All experiments were performed in triplicate and values were expressed as the Mean ± SD, n= 4 per group.

Decrease excessive inflammatory reaction and accelerate remyelination are both essential process to sensory and motor function recovery after SCI 48, 49. Numerous experiments utilizing models of macrophage depletion/ablation have reported satisfied functional and histological outcomes, suggesting that macrophages are neurotoxic and impede recovery 49. Interestingly, macrophages may also promote spinal cord repair by mediating tissue remodeling 50. These extremely different effects were explained that the different polarization states of macrophages (i.e., M1 pro-inflammatory phenotype and M2 anti-inflammatory phenotype) and suppressing M1 macrophage polarization or increasing M2 macrophage polarization could also promote injured spinal cord recovery 51. Therefore, in this study, double staining for GFAP (red) and CD68 (green) was performed to observe the quantity and distribution of M1 phenotype macrophages. As shown in Figure 5A and 5B, large amounts of M1 phenotype macrophages (CD68 positive) were observed after SCI, while the Lap/Hep@FGF4 gel treatment evidently reduced the number of M1 macrophages and prevented them from scattering; moreover, Lap/Hep gel and free FGF groups also showed a moderate effect on the number and distribution of M1 macrophages. These results demonstrated that M1 phenotype macrophages occupied both the fibrotic lesion center and the surrounding glial scar regions after SCI, whereas in Lap/Hep@FGF4 group M1 phenotype macrophages distributed almost exclusively in the glial scar region, which prevent them from spreading all over the spinal cord 52. Western blot assay of CD68, a marker of M1 phenotype macrophages, also confirmed the above results, indicating that Lap/Hep@FGF4 gel can mitigate the inflammatory response after SCI in rats (Figure 5C and 5D).

Moreover, massive researches have reported the relationship between inflammation and remyelination. The ablation of macrophages after injury such as using liposome-encapsulated clodronate would remarkably impair the clearance of myelin debris and delay remyelination 53, indicating that a moderate inflammatory reaction could promote remyelination; contrarily, both none and excessive inflammation would suppress remyelination. Here, LFB staining was performed at day 28 to investigate the effect of Lap/Hep@FGF4 gel on the myelin sheath destruction and remyelination. As illustrated in Figure 5E and 5F, the LFB-positive myelin in Lap/Hep@FGF4 treated group was prominently boosted and even across the lesion site, indicating that FGF4 loaded gel exerted a pronounced effect on myelin sheath restoration. The free FGF4 and Lap/Hep gel groups moderately reduced the myelin sheath destruction compared with SCI group. Furthermore, myelin basic proteins (MBPs) in each group were examined by Western blot. The results suggested that the MBP protein expression sharply decreased after SCI (Figure 5G and 5H) was remarkably increased by the Lap/Hep@FGF4 treatment, which further confirmed the the LFB staining results.

In summary, Lap/Hep@FGF4 could reduce the number of M1 phenotype macrophages and restrain their randomly distributing in the injured spinal cord; moreover, the process of remyelination was significantly enhanced by Lap/Hep@FGF4 treatment, suggesting that the effect of FGF4 could be boost by the Lap/Hep gel and regulate myelin debris, excessive inflammatory factors to accelerate axonal regeneration 54.

### Lap/Hep@FGF4 induced axon extension after SCI by improving microtubule stabilization and moderating the behavior of the growth cone

The capability of regeneration and reconnection with other neurons by destroyed axon is definitely difficult after spinal cord injury 55. Microtubule stabilization, a process of remodeling cytoskeleton structures, is considerable to initiate the regrowth of injured axon and regulate the migration of growth cone 15. Primarily, polarization of neurons is regulated by the microtubule stability status 56. Secondly, microtubule dynamics, which are ATP‐dependent forces on the microtubules, play a remarkable role in neurite formation and function 57. Thirdly, microtubule instability will result in retract or fragmented degenerative morphologies of injured axons 58. Therefore, dynamic microtubule is beneficial to injured axon to form a robust growth cone from severed axon stumps 59.

**Figure 6:**
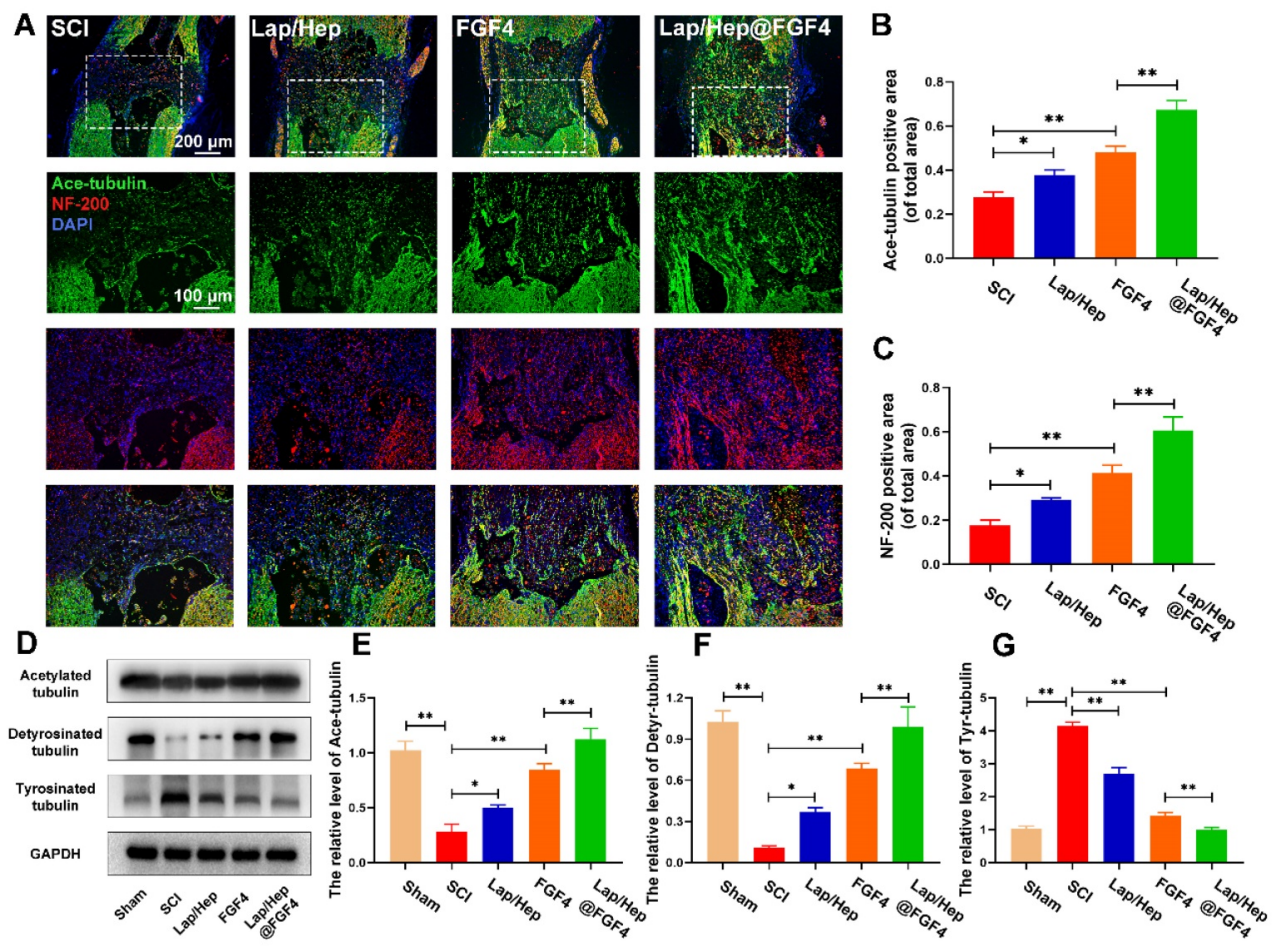
** Lap/Hep@FGF4 enhances axon regeneration through microtubule stabilization.** (A) Double immunofluorescence staining of Ace-tubulin (green) and NF-200 (red) in axons at the lesion site on the 28th day after SCI. (B, C) Quantification data of Ace-tubulin positive and NF-200 positive area in spinal cord. (D-G) Protein expression and quantification data of Ace-tubulin, Detyr-tubulin and Tyr-tubulin in each group. All experiments were performed in triplicate and values were expressed as the Mean ± SD, n= 4 per group.

To investigate whether the axonal regeneration was affected by the Lap/Hep@FGF4 gel and the mechanism of microtubule stabilization, immunofluorescence staining of acetylated tubulin (Ace-tubulin, a microtubule polymerizing protein), GFAP and neurofilaments (NF-200) were performed in all groups. As illustrated in Figure S4, there were few Ace-tubulin-positive axons at the lesion site in SCI group, whereas free FGF4 and Lap/Hep@FGF4 treatment significantly increased the number of the Ace-tubulin-labeled fibers that passed through the glial scar. Double staining of NF-200 (red) and Ace-tubulin (green) in the axons yielded similar results (Figure 6A-6C). Specifically, the Lap/Hep gel showed an inappreciable increase of Ace-tubulin/NF-200 positive axon, while the free FGF4 and Lap/Hep@FGF4 treatment group presented more Ace-tubulin-labeled fibers with more neurofilaments in the injured site. Furthermore, the protein levels of Ace-tubulin, detyrosinated tubulin (Detyr-tubulin, a microtubule depolymerizing protein) and tyrosinated tubulin (Tyr-tubulin, a microtubule depolymerizing protein) in each group were examined by Western blot (Figure 6D-6G). The results were consisted with the immunofluorescence staining, which indicated microtubule stabilization was essential for axon extension and can be enhanced by the combination of the Lap/Hep gel and FGF4.

**Figure 7:**
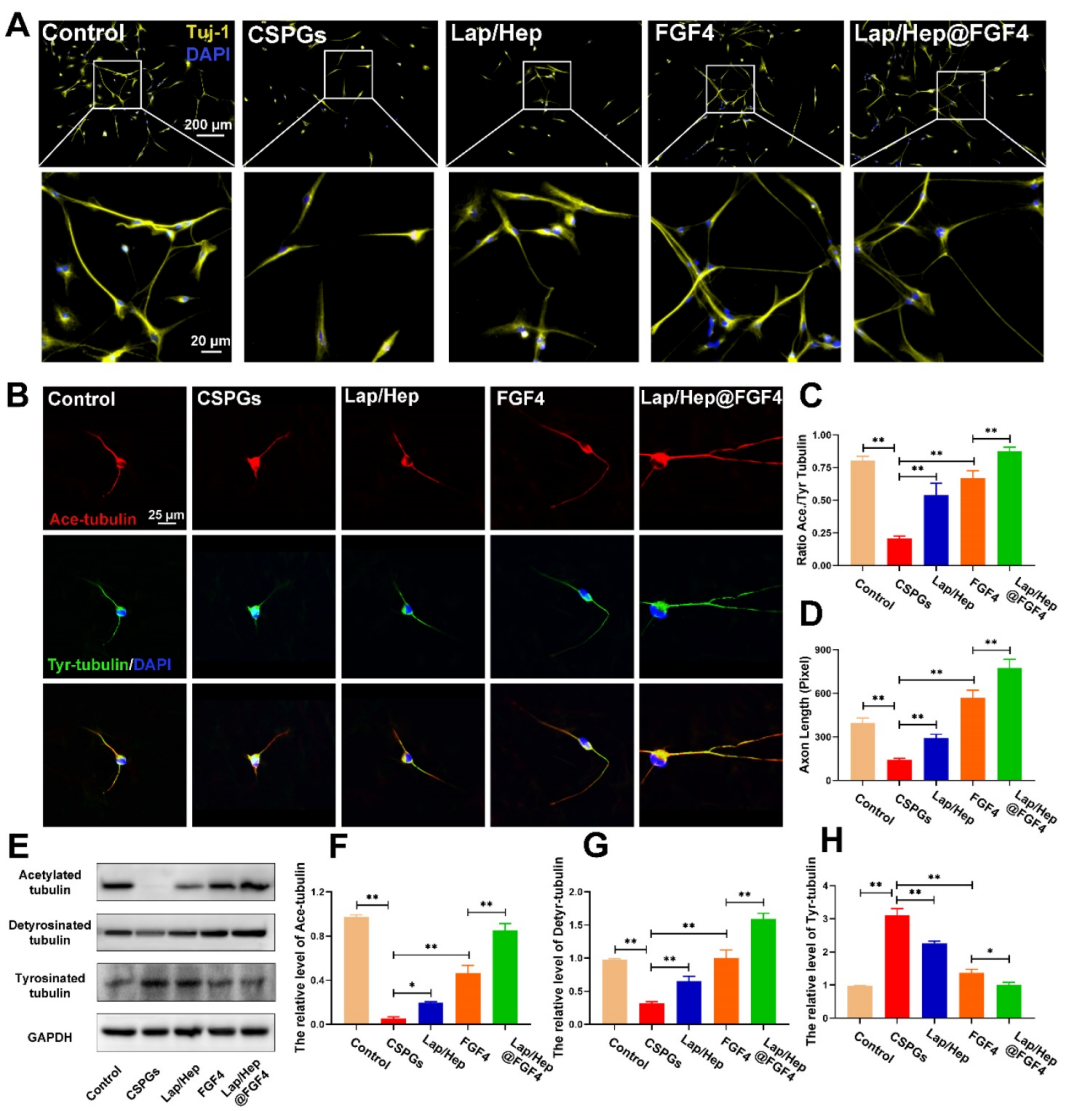
** Lap/Hep@FGF4 enhances axon formation via microtubule‐stabilization in neurons.** (A) Beta-3 tubulin (Tuj-1) immunolabeling of neurons with or without inhibitory chondroitin sulfate proteoglycans (CSPGs, 3.34 µg/mL) substrates in primary cortical neurons. (B) Co‐immunofluorescence images show Ace‐tubulin (green) and Tyr‐tubulin (red) in primary cortical neurons. (C, D) Quantification of A/T ratio and axonal length from B. (E-H) Protein expression and quantification data of Ace-tubulin, Detyr-tubulin and Tyr-tubulin in each group. All experiments were performed in triplicate and values were expressed as the Mean ± SD.

Furthermore, the mechanism of microtubule stabilization underlying the neuron axonal extending was also investigated *in vitro.* The neurons were firstly treated by the chondroitin sulfate proteoglycans (CSPGs) to suppress axonal growth, followed by the treatment with Lap/Hep gel, free FGF4 and Lap/Hep@FGF4 60. As shown in Figure 7A, the primary cortical neurons cultured with normal neurons medium performed branching morphology, while the neurons treated by CSPGs stimuli showed retraction or breakdown of axons. Interestingly, the axons of neurons could be remarkably re-extended by FGF4 and Lap/Hep@FGF4 gel treatment. Additionally, Ace- and Tyr-tubulin double immunostaining showed that FGF4 without Lap/Hep gel induced axon extension by improving microtubule stabilization *in vitro*; moreover, Lap/Hep@FGF4 treated neurons presented significantly longer axons than free FGF4 or Lap/Hep gel group (Figure 7B). Meanwhile, the expression of Ace-tubulin and the ratio of Ace to Tyr-tubulin (A/T ratio) were significantly improved compared with the CSPGs group (Figure 7C and 7D). Besides, we examined the expression of acetylated, detyrosinated and tyrosinated tubulin in each group by Western blotting and the results were consistent with the double immunostaining (Figure 7E-7H).

Collectively, the microtubule stabilization of neurons and the axon regeneration were both enhanced by FGF4 treatment, while Lap/Hep showed inappreciable effects on axon reaeration *in vitro* compare with the *in vivo* result. It could be explained that the degradation rate of Laponite is slower *in vitro* than *in vivo*; thus, the bioactive products degenerated by Laponite were inadequate for activating the cellular pathway. Moreover, the boosting effect of Lap/Hep@FGF4 gel revealed that controlled release of FGF4 combined with certain degradation products of Laponite could show a synergistic effect in microtubule stabilization.

### Lap/Hep@FGF4 affected the activity and localization of mitochondria in axon during neuron developing

**Figure 8:**
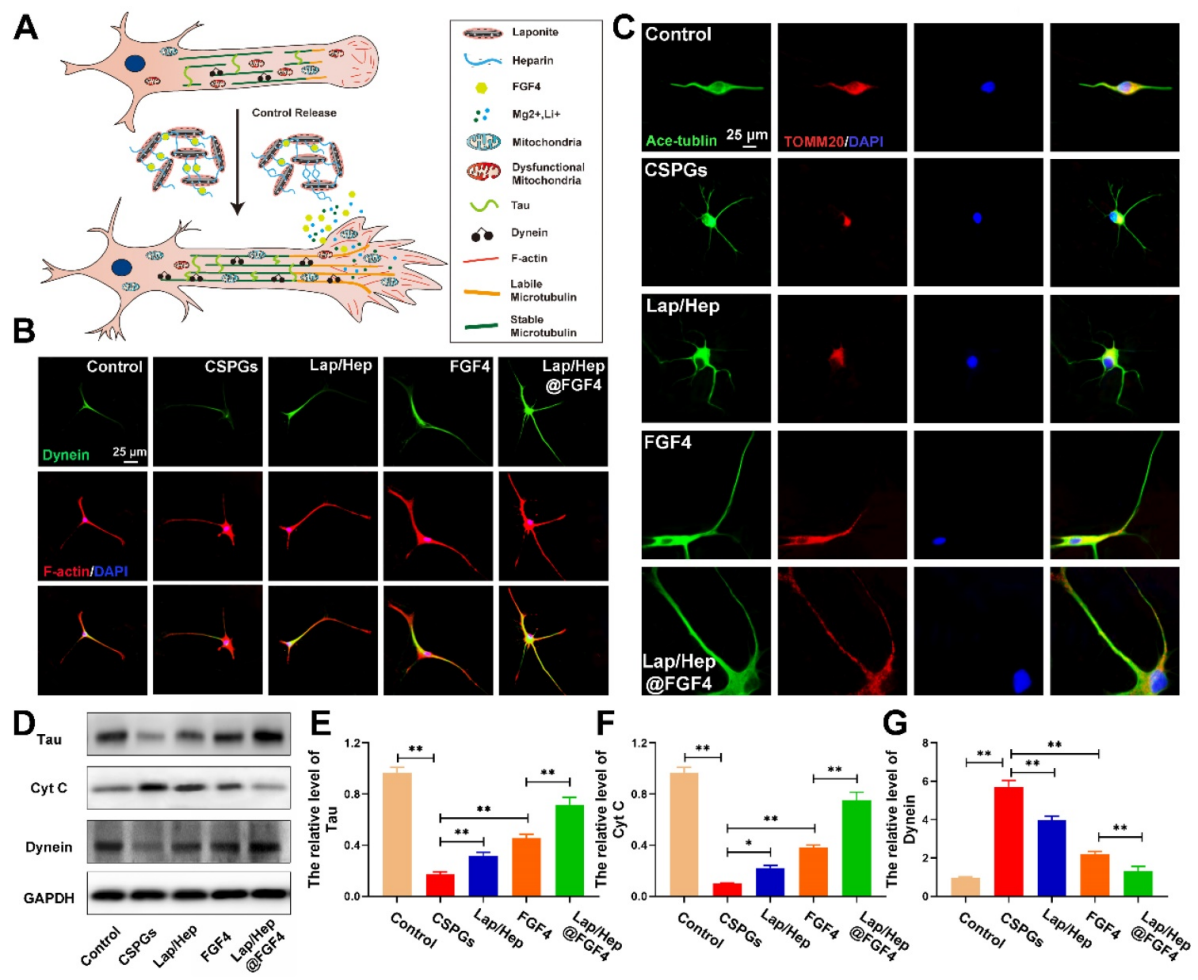
** Lap/Hep@FGF4 affects the activity and localization of mitochondria during axon migration.** (A) Schematic showing how Lap/Hep@FGF4 improves axon regeneration and the relationship between microtubule, dynein and growth cone. (B) Co-immunofluorescence images showing the distribution of dynein (green) and F-actin (red) in the growth cone in primary cortical neurons. (C) Double immunofluorescence staining show distribution of mitochondria (TOMM20, red) in axon (Ace‐tubulin, green) of primary cortical neurons in each group. (D-G) Western Blot protein expressions and quantification data of Tau, cytochrome c and Dynein in each group. All experiments were performed in triplicate and values were expressed as the Mean ± SD.

In the axon, cytoplasmic Dynein would drive bouts of coordinated and rapid transport in several microtubules even short 61. Previous researches have reported cytoplasmic Dynein combined with microtubules or actin cytoskeleton propelling short microtubules to the growth cone of neurons and form a fan‐shaped structure at the tip of the growing axon 62 (Figure 8A). To examine the relationship between Lap/Hep@FGF4 and Dynein protein, Dynein (green) and F-actin (red) immunofluorescence staining was performed (Figure 8B), which showed that the intensity of Dynein at the primary cortical neurons was higher at the axon and growth cone in Lap/Hep@FGF4 group; moreover, clearly Dynein staining can be observed at the end of the axon. Additionally, Lap/Hep@FGF4 gel induced more robust growth cones with higher intensity of F‐actin and the overlapped area among Dynein and F-actin were significantly enhanced. Therefore, consistent with previous results, Lap/Hep@FGF4 treatment showed a therapeutic effect on SCI by regulating microtubule stabilization to enhance the intrinsic growth ability of axon.

Moreover, axonal regeneration is an intricate process for injured neurons, which needs to recruit mitochondria to generate energy and interact with microtubule and microtubule‐based motor proteins for initiating growth cone and axonal extension 63. Therefore, we hypothesized that Lap/Hep@FGF4 gel could promote axonal trafficking of mitochondria to accelerate axonal regeneration underlying the mechanism of microtubules stabilization. Co‐labeled Ace-tubulin and TOMM20 (a maker of mitochondria) in primary cortical neurons was implemented to verity this assumption. As shown in Figure 8C, the mitochondria were gradually transported to the end of axon after Lap/Hep@FGF4 treatment, while the mitochondria mainly distributed around the cell nucleus in other four groups. Interestingly, the positive staining of mitochondria was significantly decreased after CSPGs stimuli, while efficiently increased and distributed along the axon after administration of Lap/Hep@FGF4.

Additionally, Tau protein, which can modulate microtubule dynamics and stabilization, was significantly increased with Lap/Hep@FGF4 treatment (Figure 8D and 8E)*.* The western blot analysis results for cytochrome c (a maker of dysfunctional mitochondria) and Dynein revealed similar differences (Figure 8D, 8F and 8G). Collectively, Lap/Hep@FGF4 may have effects on promoting mitochondrial fusion and regulating mitochondrial localization in neuronal axons, which were conducive to long-range axonal regeneration.

## Conclusions

A versatile injectable hydrogel has been designed to preserve FGF4 bioactivity and control its release. This Lap/Hep@FGF4 gel can be obtained by simply mixing the FGF4-heparin complexes and Laponite dispersion at appropriate ratio. The Lap/Hep@FGF4 hydrogel effectively enhanced the recovery of SCI when administered via in situ injection *in vivo*. Lap/Hep@FGF4 hydrogel exerted neuroprotective effects and created advantageous conditions for functional restoration due to its ability of reactive astrogliosis suppression, fibrotic scars inhibition, inflammatory reaction reduction, remyelination and axonal regeneration through increasing the level of stabilizes microtubule and activating mitochondria to unleash the intrinsic potential regrowth. Combined with the moderate regeneration effects after SCI treated by Lap/Hep gel and free FGF4 alone, Lap/Hep@FGF4 hydrogel could facilitate and prolong FGF4 delivery to the damaged spinal cord and show a synergistic effect together with Laponite. The neuroprotection benefit of this synergistic delivery system in easy preparation, quick gelation, good biocompatibility and biodegradability make it a clinically feasible therapeutic approach for patients suffering from SCI.

## Supplementary Material

Supplementary figures and tables.

## Abbreviations

SCI: spinal cord injury
FGF4: fibroblast growth factor 4
MAPs: microtubule associated proteins
BSCB: blood spinal cord barrier
ATP: adenosine triphosphate
ddH2O: double distilled water
PBS: phosphate buffered saline
LFB: luxol fast blue
BBB: Basso-Beattie-Bresnahan
MAP-2: microtubule-associated protein 2
ANOVA: one-way analysis of variance
CSPGs: chondroitin sulfate proteoglycans
ECM: extracellular matrix
MAGs: myelin-associated glycoproteins
MBPs: myelin basic proteins
