# Supplementary material for: An injectable heparin-Laponite hydrogel bridge FGF4 for spinal cord injury by stabilizing microtubule and improving mitochondrial function
Source: Theranostics. 2019 Sep 21;9(23):7016–32. doi: 10.7150/thno.37601 (PMC6815951; doi:10.7150/thno.37601)
Supplement: Supplementary file 1 — Supplementary figures and tables. [file thnov09p7016s1.pdf]

Supplementary Materials

An injectable heparin-Laponite hydrogel bridge FGF4 for spinal cord injury by stabilizing microtubule and improving mitochondrial function

Chenggui Wang[1,2], Zhe Gong[1,2], Xianpeng Huang[1,2], Jingkai Wang[1,2], Kaishun Xia[1,2], Liwei Ying[1,2], Jiawei Shu[1,2], Chao Yu[1,2], Xiaopeng Zhou[1,2], Fangcai Li[1,2,*], Chengzhen Liang[1,2,*] and Qixin Chen[1,2,*]

[1] Department of Orthopedics Surgery, 2nd Affiliated Hospital, School of Medicine, Zhejiang University, 88 Jiefang Road, Hangzhou, 310009, Zhejiang, People's Republic of China

[2] Department of orthopedics Research Institute of Zhejiang University, Hangzhou, Zhejiang, People's Republic of China

[*] Corresponding author: Qixin Chen (zrcqx@zju.edu.cn), Chengzhen Liang (liangchengzhen@zju.edu.cn), Fangcai Li (lifangcai@zju.edu.cn)

Material and Methods

Molecular modeling

FGF4 (PDB ID: 1IJT) was chosen for docking studies. The protein was prepared for docking after being downloaded from PDB (https://www.rcsb.org/). After being minimized using PyMoL (version 1.7.6), the lowest energy conformations for docking were determined via default parameters. The protein-ligand docking analysis was conducted using AutoDockTools (version 1.5.6), which can provide ligand binding flexibility with the binding pocket residues. The images were finally generated using UCSF PyMoL.

Primary cortical neuron, astrocyte cultures and drug treatment

Primary cortical neurons were dissociated from 2-day-old neonatal Sprague-Dawley (SD) rats. Briefly, the cerebral cortex was dissected to small pieces, transferred into a tube and digested in TrypLE Select (Gibco, USA) solution at 37 $^{\circ}$C for 10 min, followed by centrifugation at 1,000 rpm for 5 min. The cell suspension was first cultured on poly-D-Lysine precoated glass coverslips in DMEM medium (Invitrogen, Carlsbad, California) with 10% (vol/vol) FBS and 10% (vol/vol) F12. After 4 h, culture medium was replaced by Neurobasal (Stem cell technology, USA) medium containing 2% B27 (Gibco, USA) and 1% L-glutamine (Sigma-Aldrich, USA). To evaluate the effects of FGF4 and hydrogels on microtubule dynamics and mitochondria function, primary cortical neurons (approximately 1×10^5 cells/well) were re-plated and cultured in 12-well plates with or without 3.34 μg/mL CSPGs (chondroitin sulfate proteoglycans, Sigma-Aldrich, USA) for 48 h. Then 100 μL hydrogels (Lap/Hep or Lap/Hep@FGF4 containing 100 ng FGF4) or free 100 ng FGF4 solution were added into the transwell inserts (pore size 0.4 mm) and co-incubated for 72 h. After that, cells were used for western blot or fixed with 4% paraformaldehyde and prepared for Tuj-1, ace-tubulin, tyr-tubulin, Dynein, TOMM20 and F-actin immunofluorescent staining.

Purified primary astrocytes were also obtained from neonatal SD rats. In brief, after removal of the meninges, brain tissue was minced and incubated at 37 $^{\circ}$C for 30 min in DMEM medium in the presence of 0.25% trypsin (Sigma-Aldrich, USA) and 300 g/mL DNase I (Sigma-Aldrich, USA). The dissociated cells were triturated with 0.25% FBS and centrifuged at 300 g for 5 min. Following dilution with astrocyte-specific medium (DMEM containing 10% FBS, 0.2 mM L-glutamine, and 1% penicillin-streptomycin), the cells were then placed on culture dishes. After

7-10 days culture in a humidified CO_2 incubator at 37 $^{\circ}$C, the astrocytes were ready and used for experiments. To investigate the influence of FGF4 or hydrogels on migration and polarization of primary astrocytes (about 1×10^5 cells/well) were transferred into 12-well plates. Then 100 μL hydrogels (Lap/Hep or Lap/Hep@FGF4 containing 100 ng FGF4) or free 100 ng FGF4 solution were added into the transwell inserts (pore size 0.4 mm). After that, cells were used for wound starch assay or co-incubated for 72 h, fixed with 4% paraformaldehyde and prepared for *in vitro* F-actin immunofluorescent staining.

Wound scratch assay

A wound scratch assay with a transwell system was performed to investigate the migratory activity of primary astrocytes. Cells were seeded into a 12-well plate at a density of 1×10^5 cells/well. After 24 h of incubation, a scratch was made in the cell monolayer to create a wound gap using a 200 μL pipette tip, and then 100 μL hydrogels (Lap/Hep or Lap/Hep@FGF4 containing 100 ng FGF4) or free 100 ng FGF4 solution were added into the transwell inserts. Wound scratch was observed and pictured at 0, 1, 2, 3, 4 and 5 days under inverted microscope at 100× magnification (Olympus, Tokyo, Japan). The wound scratch images were analyzed using ImageJ software (National Institutes of Health).

Flow cytometry assay

Cell viability was detected after neurons were stained by an Annexin V-FITC/PI Detection Kit (Solarbio, Beijing, China) following the manufacturer's instruction. Briefly, primary cortical neurons were treated by different hydrogels as above mentioned. After that, cells were washed by cold phosphate buffered saline (PBS) and harvested by 0.25% Trypsin with 0.02% EDTA, then centrifuged at 1000 rpm for 5 min and re-suspended in binding buffer. Then 5 μL Annexin V-FITC was added and incubated for 15 min in dark, followed by adding 5 μL PI to the flow tubes before analyzed. The flow cytometry analysis was carried out using the BD FACSCalibur flow cytometer (BD Biosciences, San Jose, USA) and analyzed by the Flowjo software.

Histological assay

Tissue preparation

Animals were anesthetized with 1% (w/v) pentobarbital sodium (4 mL/kg, i.p.) at specific time points after SCI. 1 cm section of spinal cord tissues were dissected, fixed by 4% paraformaldehyde for 6 h and processed for paraffin-embeding. Longitudinal or transverse sections (5 μm thick) were mounted on slides for following staining.

Hematoxylin and eosin staining

For H&E staining, the longitudinal or transverse sections were dewaxed and washed with PBS. Then, sections were fixed with acetone for 2 min, washed with water for 1-2 s, and stained with hematoxylin for 5 min at room temperature. After washing, the slides were immersed in 1% acidic alcohol for 5-10 s and rinsed twice (2 min per rinse) with distilled water. Eosin solution was added to re-stain the sections for 1-2 min, followed by washing and dehydrating through a gradient ethanol series (80%, 90% and 100%; 30 s per step). Finally, the sections were immersed in xylene I (5 min) and xylene II (5 min) and sealed with neutral gum. The slides were observed and images were acquired under a light microscopy (Olympus, Tokyo, Japan).

Luxol fast blue staining

For luxol fast blue (LFB) staining, the longitudinal sections were dewaxed followed by washed with PBS. The sections were immersed in 0.1% LFB at room temperature overnight, washed and de-stained with 0.05% lithium carbonate solution for 5 min, followed by dehydration with a graded series of ethanol (70% 10 s, 95% 25 s, 100% 25 s). Finally, the slides were hyalinized with xylene and mounted with neutral gum. The images were also acquired under a light microscopy (Olympus, Tokyo, Japan).

Immunofluorescence

For immunofluorescence, sections were performed with antigen retrieval in 0.01 M citrate buffer solution (pH = 6.0) after deparaffinization. Then, longitudinal sections were treated with primary antibodies targeting the following proteins: NF-200 (1:2000, Abcam), GFAP (1:2000, Abcam), microtubule-associated protein 2 (MAP-2, 1:300, Cell Signaling Technologies), Laminin (1:400, Abcam) and Ace-tubulin (1:500, Abcam). The transverse sections were treated with primary antibodies targeting GFAP (1:2000, Abcam) and CD68 (1:400, Abcam). The slides were washed four times with PBST and incubated with Dylight 488, Dylight 550 or Dylight 594 donkey anti-rabbit/mouse/goat secondary antibodies for 1 h at 37 °C. Afterwards, the sections were washed with PBS, incubated with DAPI for 7 min, rinsed with PBS and finally sealed with a

coverslip. All the images were captured by a fluorescence microscope (Leica, Wetzlar, Germany). The results of immunofluorescence staining were quantified by mean fluorescence density with ImageJ software (National Institutes of Health, Bethesda, MD, USA).

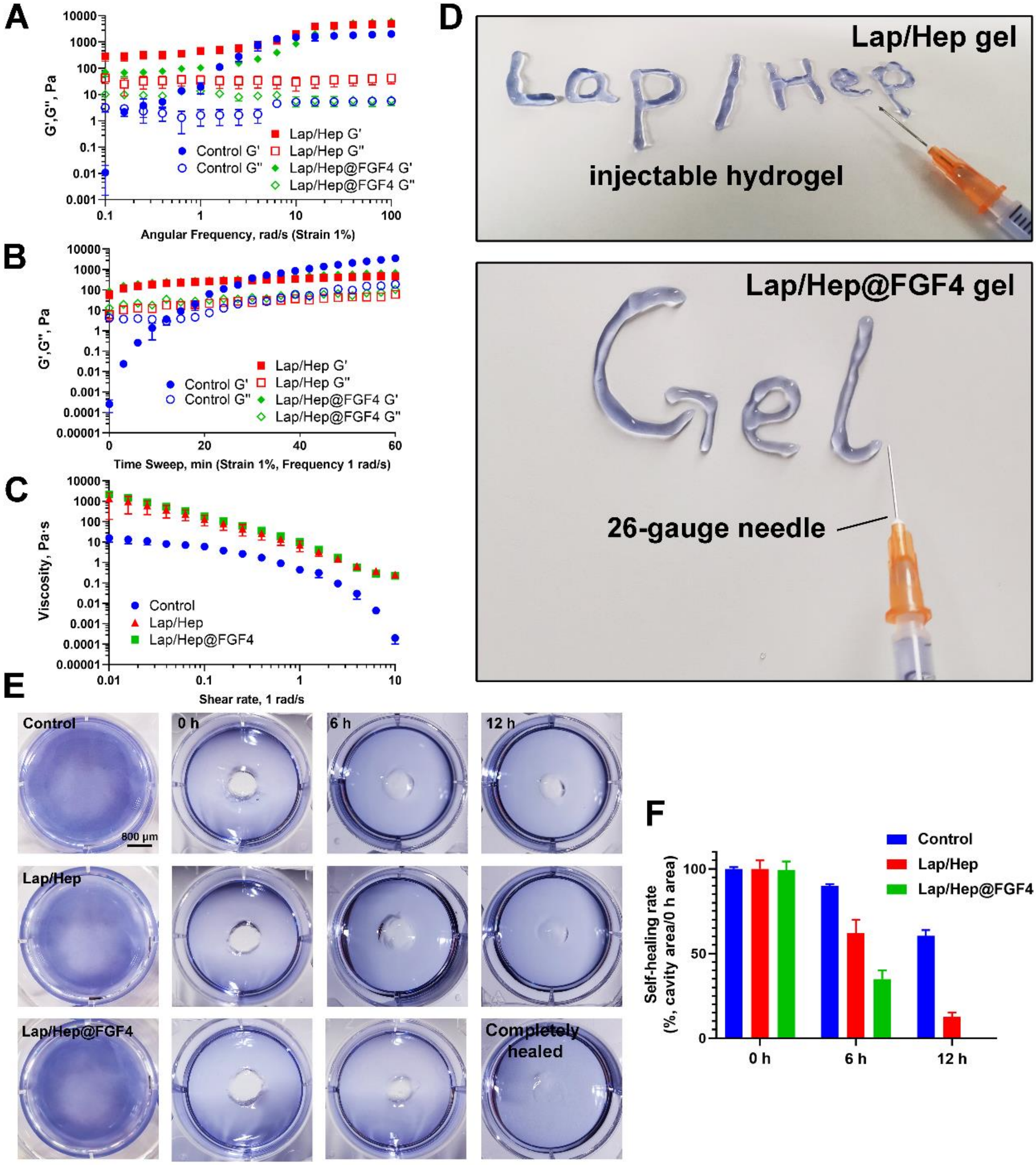

Figure S1 The viscosity of Laponite, Lap/Hep and Lap/Hep@FGF4 hydrogels. (A, B) Angular frequency sweep and oscillatory time sweep measurements of Laponite, Lap/Hep and Lap/Hep@FGF4 gels. (C) Viscosity change of Laponite, Lap/Hep and Lap/Hep@FGF4 hydrogels. (D) The Lap/Hep and Lap/Hep@FGF4 hydrogels can pass through a 26-gauge needle without clogging. (E) Gross appearance of Laponite, Lap/Hep and Lap/Hep@FGF4 hydrogels. Gels were stained with trypan blue for observation. (F) Quantification results of the self-healing rate of

Laponite, Lap/Hep and Lap/Hep@FGF4 hydrogels. All the gels show shear thinning properties, injectability and self-healing. All experiments were performed in triplicate and values were expressed as the Mean ± SD.

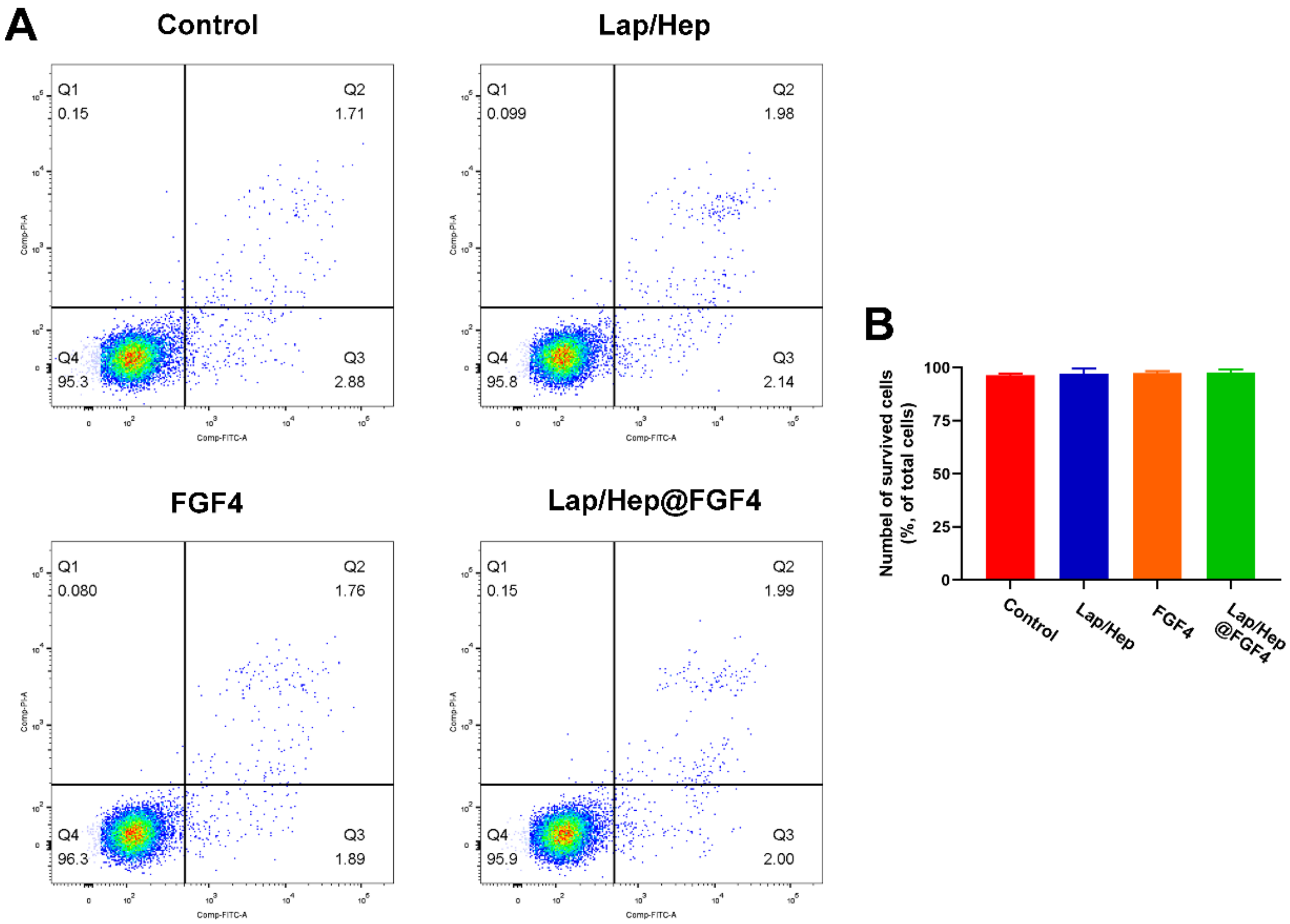

Figure S2 Flow cytometry assay the cytotoxicity of Lap/Hep@FGF4. (A) The survival rate of primary cortical neurons with treatment of Lap/Hep, free FGF4 or Lap/Hep@FGF4 using PI/annexin V-FITC staining (H) Quantification results of the survival rate of primary cortical neurons from A. All experiments were performed in triplicate and values were expressed as the Mean ± SD.

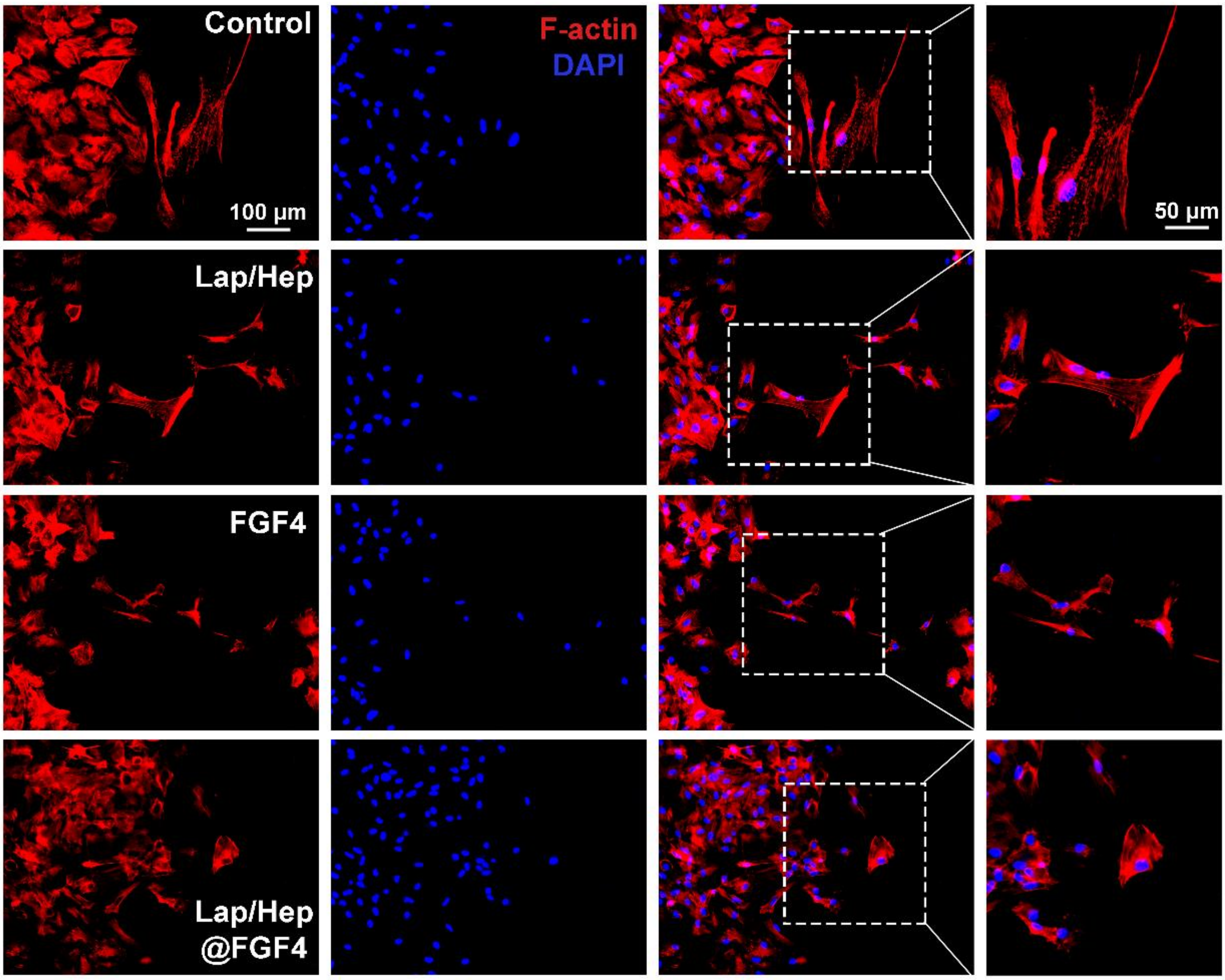

Figure S3 Lap/Hep@FGF4 suppresses the polarization of astrocytes. Immunofluorescence staining F-actin (red) show the polarization state of primary cortical astrocytes in each group. All experiments were performed in triplicate.

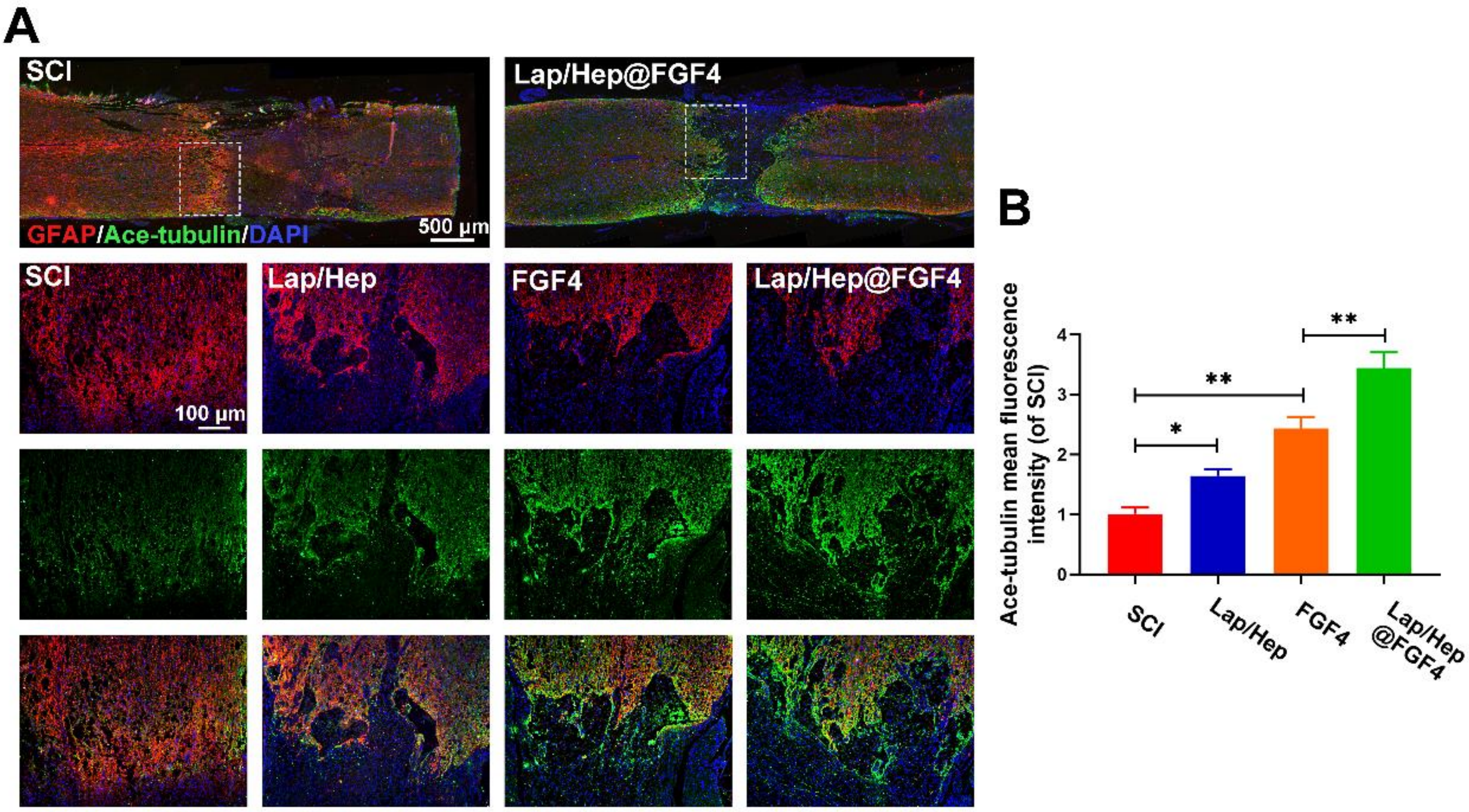

Figure S4 Lap/Hep@FGF4 improves microtubule stabilization. (A) Double immunofluorescence staining of Ace-tubulin (green) and GFAP (red) at the lesion site on the 28th day after SCI. (B) Quantification the mean fluorescence of Ace-tubulin in spinal cord. All experiments were performed in triplicate and values were expressed as the Mean ± SD.
